# JustDeepIt: Software tool with graphical and character user interfaces for deep learning-based object detection and segmentation in image analysis

**DOI:** 10.3389/fpls.2022.964058

**Published:** 2022-10-06

**Authors:** Jianqiang Sun, Wei Cao, Takehiko Yamanaka

**Affiliations:** Research Center for Agricultural Information Technology, National Agriculture and Food Research Organization (NARO), Tsukuba, Japan

**Keywords:** Deep learning, image recognition, object detection, instance segmentation, leaf segmentation, plant segmentation, graphical user interface

## Abstract

Image processing and analysis based on deep learning are becoming mainstream and increasingly accessible for solving various scientific problems in diverse fields. However, it requires advanced computer programming skills and a basic familiarity with character user interfaces (CUIs). Consequently, programming beginners face a considerable technical hurdle. Because potential users of image analysis are experimentalists, who often use graphical user interfaces (GUIs) in their daily work, there is a need to develop GUI-based easy-to-use deep learning software to support their work. Here, we introduce JustDeepIt, a software written in Python, to simplify object detection and instance segmentation using deep learning. JustDeepIt provides both a GUI and a CUI. It contains various functional modules for model building and inference, and it is built upon the popular PyTorch, MMDetection, and Detectron2 libraries. The GUI is implemented using the Python library FastAPI, simplifying model building for various deep learning approaches for beginners. As practical examples of JustDeepIt, we prepared four case studies that cover critical issues in plant science: (1) wheat head detection with Faster R-CNN, YOLOv3, SSD, and RetinaNet; (2) sugar beet and weed segmentation with Mask R-CNN; (3) plant segmentation with U^2^-Net; and (4) leaf segmentation with U^2^-Net. The results support the wide applicability of JustDeepIt in plant science applications. In addition, we believe that JustDeepIt has the potential to be applied to deep learning-based image analysis in various fields beyond plant science.

## 1 Introduction

Over the past decade, remarkable advances have been made in image analysis based on deep learning in various fields (Jiang and Li, 2020; Ben Yedder et al., 2021; Liu and Wang, 2021). In practical applications in plant science field, deep learning for image analyses has been applied at different scales. For example, at the field scale, studies such as high-throughput phenotyping and yield prediction via images captured by drones or hyperspectral cameras are hot topics (Kamilaris and Prenafeta-Boldú, 2018; Jiang and Li, 2020). On an individual scale, studies including species classification, crop disease detection, and weed detection are well researched (Christin et al., 2019; Hasan et al., 2021; Liu and Wang, 2021). In addition, at the cell level, studies such as cell type identification and stomata identification via microscopic images have been performed (Moen et al., 2019; Zhu et al., 2021). The increased availability of these techniques in various fields enhances the importance of the roles they are expected to play in the future.

Image analysis based on deep learning can be roughly categorized into three main tasks: object classification, object detection, and instance segmentation. Object classification determines the class of an object in an image. Object detection specifies the types of objects in an image and their locations, generally through bounding boxes (i.e., rectangular delimiting areas). Instance segmentation selects a pixel-wise mask for each object in an image. As images generally contain multiple objects, object detection and instance segmentation have broader practical applications than object classification. In addition to instance segmentation, salient object detection is often used to detect the primary object in an image at the pixel level (Wang et al., 2017; Borji et al., 2019). It can also be applied to one-class instance segmentation for background removal, leaf segmentation, and root segmentation.

Applying machine learning models to images captured under conditions different from those of images captured for model training degrades the inference performance. This is called the frame problem in machine learning (Ford and Pylyshyn, 1996). It is impossible to solve this problem without collecting training images under all conditions. Hence, most practical applications restrict the usage conditions to ensure that the frame is not exceeded by limiting the target objects, shooting conditions, or by other means. In scientific studies, this problem is addressed through models devised for specific projects instead of previously developed models.

Python programming language and its libraries PyTorch (Paszke et al., 2019), MMDetection (Chen et al., 2019), and Detectron2 (Wu et al., 2019), have facilitated image analysis using deep learning. However, programming experience and machine learning expertise are required to implement complicated neural networks (i.e., deep learning models) for object detection and instance segmentation tasks.

Given the required programming skills or machine learning expertise, the application of deep learning remains challenging for most beginners. Many experimentalists working full-time on wet experiments use graphical user interfaces (GUIs) in their daily work. In contrast, informatics researchers use character user interfaces (CUIs) because it is easy to perform large-scale experiments under different parameter combinations owing to the scalabilities of CUIs. Thus, suitable software should be supported on GUIs and CUIs to serve various users, making it advantageous for experimental and informatics researchers to use the same software and conduct collaborative research. Nevertheless, most existing open-source GUI-based software for deep learning-based image analysis only supports segmentation or is intended for specific purposes. For instance, RootPainter (Smith et al., 2022) and DeepMIB (Belevich and Jokitalo, 2021) support biological image segmentation using U-Net (Ronneberger et al., 2015). ZeroCostDL4Mic (von Chamier et al., 2021) implements You Only Look Once version 2 (YOLOv2) (Redmon and Farhadi, 2017) and U-Net for object detection and instance segmentation against microscopy images. Maize-IAS (Zhou et al., 2021) partially uses a faster region-based convolutional neural network (Faster R-CNN) (Ren et al., 2017) for leaf segmentation and leaf counting of maize images captured under the controlled environment. Moreover, most solutions are focused on GUIs but neglect CUIs, thus hindering expansion on the user side. Therefore, there is a high demand for image analysis software based on deep learning supporting both easy-to-use GUIs and high-scalability CUIs, to satisfy a diverse user base.

We developed the JustDeepIt software supporting GUI and CUI to train models and perform inference for object detection, instance segmentation, and salient object detection. JustDeepIt can be applied to many biological problems, such as wheat head detection, plant segmentation, and leaf segmentation. In addition, it provides an intuitive solution for biologists lacking programming experience and machine learning expertise, simplifying implementation compared with conventional programming schemes.

## 2 Method

**Figure 1:**
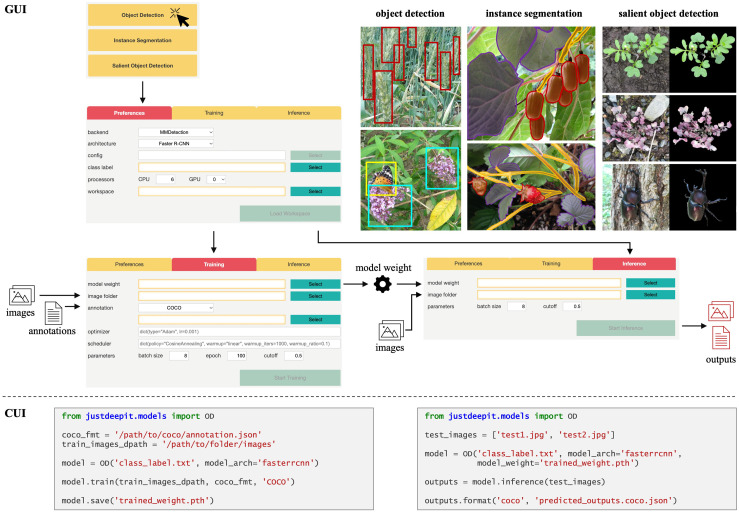
Overview of GUI and CUI implementation of JustDeepIt. For GUI usage, four main screens are implemented to select the analysis task, set standard parameters (e.g., neural network architecture, temporary folder), train a model, and infer new images with the trained model, respectively. For CUI usage, import the modules based on analysis tasks and then use the initialization method (e.g., **OD**), **train, save, and inference** methods to set up a model, train the model, save the model, and infer new images with the trained model, respectively.

JustDeepIt is implemented using Python and is easy to interoperate with various Python packages. It provides GUI and CUI for deep learning-based image analysis, including object detection, instance segmentation, and salient object detection (**Figure 1**). The source code is deposited in GitHub at https://github.com/biunit/JustDeepIt under an MIT License. An overview of implementations of the user interfaces and main functions of JustDeepIt are described in the following subsections. Detailed documentation, including installation guides and tutorials, is available on the project website.

### 2.1 Implementation of user interfaces

The GUI of JustDeepIt is implemented using FastAPI (Ramírez, 2018), a straightforward Python library for building simple web applications. It allows deep learning-based image analysis tasks with simple mouse and keyboard operations without writing codes (**Figure 1**). Upon launching the GUI, the user is prompted to select an analysis task; after this selection, the analysis screen is displayed. The analysis screen has three tabs, *Preferences*, *Training*, and *Inference*. The *Preferences* tab allows users to set standard parameters for training and inference, such as neural network architecture, class labels (object names targeted for detection or segmentation), a temporary directory to save the intermediate and final results, and more. The *Training* and *Inference* tabs are used to train a model by specifying training images and the corresponding annotations and inferring new images with the trained model, respectively.

The CUI of JustDeepIt can be used via application programming interfaces (APIs). The most complicated procedures (e.g., data registration, model initialization, output adjustment) are encapsulated into the APIs containing a few intelligible functions to simplify usage. The three main API functions are **train** for training detection or segmentation models, **save** for saving the trained model weights, and **inference** for detection or segmentation on test images. Example codes for training object detection models and using the model for inference are shown in **Figure 1**. Additional usage examples (e.g., building a web application) and arguments of these functions are available on the project website.

### 2.2 Object detection and instance segmentation

Object detection and instance segmentation models in JustDeepIt are internally built based on the MMDetection (Chen et al., 2019) or Detectron2 (Wu et al., 2019) libraries. The user can choose MMDetection or Detectron2 as the backend to build the corresponding models. JustDeepIt supports various well-known neural network architectures. For object detection, Faster R-CNN (Ren et al., 2017), YOLOv3 (Redmon and Farhadi, 2018), Single-Shot Multibox Detector (SSD) (Liu et al., 2016), and RetinaNet (Lin et al., 2018) are available to meet different user needs. For instance segmentation, Mask R-CNN (He et al., 2017) is available. Furthermore, JustDeepIt allows user-customized neural network architectures to accommodate users who wish to use architectures that are not implemented in the software. For example, Faster R-CNN implemented in JustDeepIt uses VGGNet (Simonyan and Zisserman, 2015) for feature extraction during the detection process; the users may want to change VGGNet to other architectures such as ResNet (He et al., 2015). To accomplish this, users can (i) either download already-prepared architecture configuration files from MMDetection or Detectron2 GitHub repositories or create their own configuration file from scratch and then (ii) input the configuration file into JustDeepIt to build a model for training and inference.

For model training, JustDeepIt requires image annotations in the Common Objects in Context (COCO) format, which can be generated using GUI-based free software such as COCO Annotator (Brooks, 2021) and Computer Vision Annotation Tool (Boris et al., 2021). After the user specifies the location of the image dataset and corresponding annotations through the *Training* tab of GUI or the training function of CUI, JustDeepIt uses them to build the related model.

For object detection using the trained model, the user can specify the trained weights and folder containing the test images for detection through the *Inference* tab of GUI or the inference function of CUI. For GUI usage, the inference results are automatically saved as images with bounding boxes or contour lines around the detected objects and a JSON file of the inference results in COCO format. For CUI usage, the user can specify whether the inference results should be saved as annotated images or an annotation file.

### 2.3 Salient object detection

The module for salient object detection in JustDeepIt is based on U^2^-Net (Qin et al., 2020) and written using the PyTorch library. The GUI and the training and detection functions processing are similar to those used for object detection.

**Figure 2:**
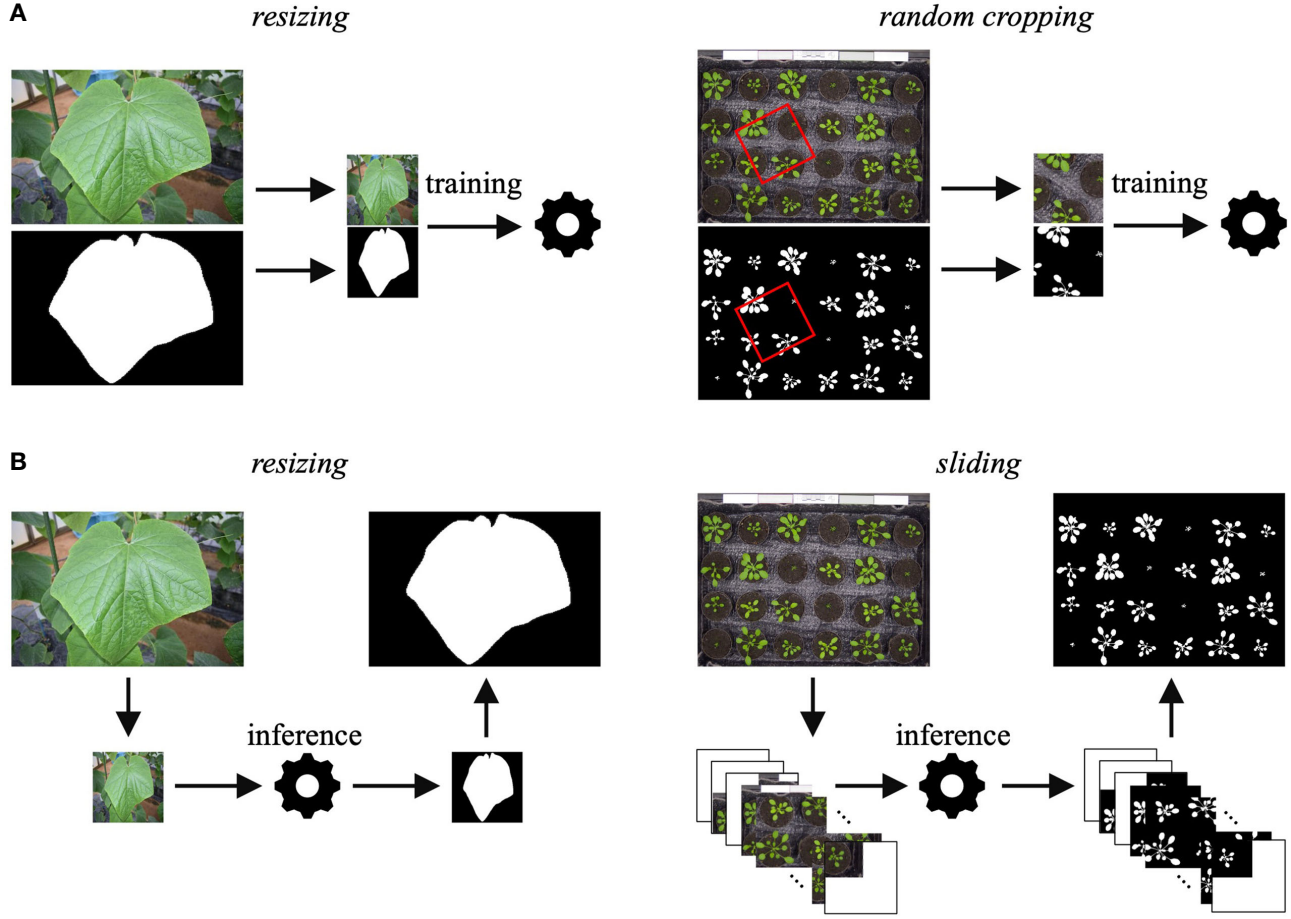
**(A)** Training approaches of *resizing* and *random cropping* are implemented in JustDeepIt. **(B)** Inference approaches of *resizing* and *sliding* (corresponding to training using *resizing* and *random cropping*, respectively) are implemented in JustDeepIt.

JustDeepIt requires training images with annotations (either COCO format annotations or mask images) for model training. Although the U^2^-Net implementation for JustDeepIt requires images of 288 × 288 pixels as the canonical input, images of various sizes are captured for applications in plant science. Thus, JustDeepIt provides two approaches for training on images of various sizes: *resizing* and *random cropping* (**Figure 2A**). *Resizing* changes the image resolution to 288 × 288 pixels for training in U^2^-Net. This approach is suitable for images containing a few large target objects. In contrast, *random cropping* randomly selects small areas of *p* × *p* pixels at random angles from the original images, where *p* can be specified according to the complexity of the intended images and tasks. The images of *p* × *p* pixels are then resized to 288 × 288 pixels for training. This approach is suitable for images containing many small target objects and details.

JustDeepIt implements *resizing* and *sliding* for salient object detection (**Figure 2B**). *Resizing* changes the scale of the input images to 288× 288 pixels for detection and then restores the detection result to the input size. *Sliding* first crops square areas of *p* × *p* pixels from the top left to the bottom right of the image step-by-step, where *p* can be specified by the user. It then resizes the areas to 288 × 288 pixels to perform salient object detection in each area. Finally, it merges the processed areas into a single image. In addition to salient object detection, summarization functions (e.g., counting the number of objects in the image, quantifying colors, measuring the area of each object) are available in JustDeepIt.

## 3 Results

We prepared four case studies as practical examples of JustDeepIt and reported the results in this section. Detailed procedures for these case studies can be found in the JustDeepIt documentation.

### 3.1 Wheat head detection

We show an example of JustDeepIt performing object detection for the wheat head, a prevalent task in plant science. The global wheat head detection (GWHD) dataset, containing 4700 images of 1024 × 1024 pixels for wheat head detection evaluation (David et al., 2020), was used in this case study. We randomly selected 80% of the images in the GWHD dataset for training and used the remaining 20% for validation. We constructed Faster R-CNN, YOLOv3, SSD, and RetinaNet with MMDetection backend and Faster R-CNN and RetinaNet with Detectron2 backend for training and validation with the GWHD dataset. To initialize each architecture, we retrieved the pretraining weights from the GitHub repositories of MMDetection and Detectron2.

The training was performed for 100 epochs with a batch size of eight and an initial learning rate of 0.0001. Validation was performed using the trained architectures against the validation images, and the mean average precision (mAP) was calculated from the validation results. Training and validation were independently repeated five times to mitigate the influence of randomness. These processes were executed on an Ubuntu 18.04 system equipped with an NVIDIA Tesla V100 SXM2 graphics processor, an Intel Xeon Gold 6254 processor, and 64 GB of memory.

**Figure 3:**
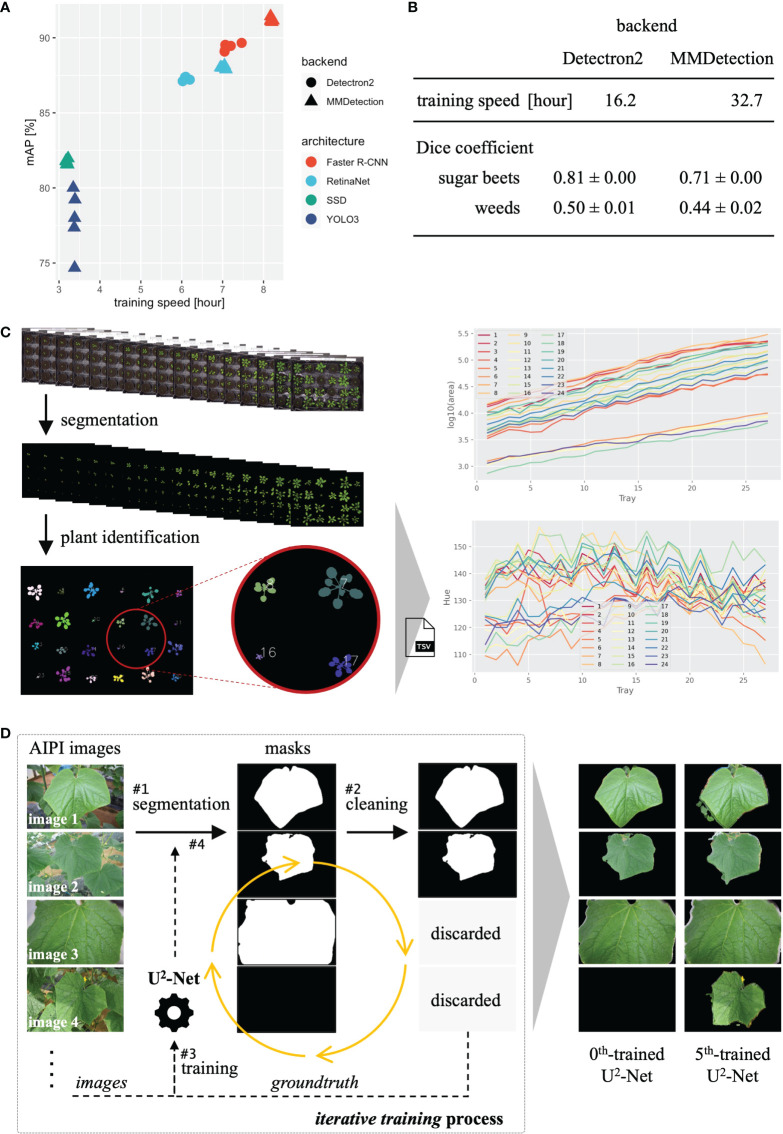
Results of three case studies. **(A)** Training time and validation mAP of object detection. **(B)** Training time and validation mAP of instance segmentation. **(C)** The process and analysis result (projected area and color hue) of plant segmentation with time-series plant images. **(D)** Processes and sample results of iterative training of U^2^-Net for leaf segmentation.

For the five training and validation runs, Faster R-CNN provided a relatively high validation mAP with a relatively slow training speed, YOLOv3 and SSD provided lower mAP with faster training speed, and RetinaNet provided intermediate mAP and training speed (**Figure 3A**). In addition, the Faster R-CNN and RetinaNet with MMDetection backend provided slower training and higher validation mAP than those with the Detectron2 backend. Hence, different backends and neural network architectures provided distinct performances and training speeds, and users should select the appropriate backend and architecture according to the application.

### 3.2 Sugar beets and weeds segmentation

To represent the case study of instance segmentation with JustDeepIt, we performed weed and crop (here, sugar beet) segmentation, which is one of the tasks in high-demand in the agriculture sector, on the SugarBeets2016 dataset (Chebrolu et al., 2017). The SugarBeets2016 dataset has 11,552 RGB images captured under fields and the annotations for sugar beets and weeds. We randomly selected 5,000 and 1,000 images for training and validation, respectively. We constructed Mask R-CNN with MMDetection and Detectron2 backends for training and validation. We retrieved the pretraining weights from the GitHub repositories for initializing each architecture, as in subsection 3.1.

Training and validation were performed using the same parameters, processes, and system devices as in subsection 3.1, except that the batch size was set to four. The Dice coefficient was calculated from the validation results. For the five training and validation runs, Mask R-CNN with Detectron2 backend provided relatively higher Dice coefficients and faster training speed than that with MMDetection backend (**Figure 3B**). As in the case of object detection, the result was well characterized by the backend.

### 3.3 Plant segmentation with U^2^-Net

As a case study of salient object detection with JustDeepIt, we use the Plant Phenotyping Dataset, a popular benchmark dataset for plant segmentation (Minervini et al., 2015; Minervini et al., 2016). The dataset contains 27 images of 3108 × 2324 pixels, and each image contains 24 individual plants. We used U^2^-Net implemented in JustDeepIt for plant segmentation. We randomly selected three images to train U^2^-Net using the *random cropping* approach. The training was performed with the default parameters in a macOS Big Sur system equipped with a Quad-Core Intel Core i5 processor (2.3 GHz) and 16 GB of memory. The training took approximately 6.5 hours with four processors but without any graphics processor.

Salient object detection was performed using the trained U^2^-Net with the *sliding* approach for the 27 images with default parameters. Detection took approximately 2.3 hours, as the 27 images (3108 × 2324 pixels) were sliced into 27 × 130 small images (320 × 320 pixels) by the *sliding* approach, requiring substantial processing time. In addition, as the 27 images show time-series data, we summarized object statistics (e.g., projected area, plant color) over time (**Figure 3C**). The result indicates that the areas of the plants increased, and their colors varied over time.

### 3.4 Iterative training of U^2^-Net for improved leaf segmentation

Extracting salient objects by removing the background may improve the performance of image analysis and can be applied to various image analysis tasks. As another case study for salient object detection, we trained U^2^-Net for leaf segmentation on the Pest Damage Image Dataset (National Agriculture and Food Research Organization (NARO), 2021). The dataset comprises images of four crops, including cucumber tagged by disease or pest names, whereas no annotations of bounding boxes or segmentation are available. Here, we proposed iterative training to U^2^-Net using unannotated images for leaf segmentation for cucumber.

Iterative training proceeded as follows (**Figure 3D**). In step 1, we used U^2^-Net trained on the DUTS dataset (Wang et al., 2017) (0^th^-trained U^2^-Net) obtained from the corresponding GitHub repository (Qin et al., 2021) for leaf segmentation of cucumber leaf images. In step 2, the images whose nearly complete area was detected as a salient object (image 3 in **Figure 3D**) or without detection (image 4 in **Figure 3D**) were discarded. In step 3, we used the remaining images and detection results (i.e., mask images) to retrain U^2^-Net. In step 4, we used the trained U^2^-Net from step 3 to perform salient object detection for the cucumber leaf images again. Then, we repeated steps 2–4 to retrain U^2^-Net five times, obtaining the 5^th^-trained U^2^-Net, and training was performed using the CUI for efficiency.

The 0^th^-trained U^2^-Net failed to detect leaves in images containing multiple leaves. In contrast, the 5^th^-trained U^2^-Net successfully detected the main salient leaf in every image (**Figure 3D**). Thus, even without annotations, we built a model for leaf segmentation using existing techniques. General users can use such approaches *via* the simple JustDeepIt API to extend the range of applications.

## 4 Discussion

The widespread use of deep learning technologies is gradually contributing to various scientific fields. Thus, it is vital to support the ease of technology usage for everyone, regardless of their research backgrounds and programming skills. As experimental researchers use GUIs and informatics researchers use CUIs mostly, developing software, which supports GUIs and CUIs and is not restricted to any specific tasks, is essential.

In the field of plant sciences, various GUI-based software, implemented for deep learning-based image analysis, has been developed. However, most software implementing one or a few neural network architectures to solve specific problems and only support GUI (Belevich and Jokitalo, 2021; von Chamier et al., 2021; Smith et al., 2022; Zhou et al., 2021). In contrast, JustDeepIt was developed to fill these gaps, enabling various image analysis tasks using deep learning technologies in a single software. JustDeepIt implements multiple neural network architectures suitable for different application scenarios and supports both GUI and CUI, increasing flexibility according to the task. As shown in our case studies with JustDeepIt, GUI is ideal for building models from available data effortlessly, and CUI is suitable for facilitating the use of a model as an extension (e.g., iterative training of U^2^-Net). Furthermore, we believe that by supporting both GUI and CUI, collaborative research between experimental and informatics researchers can proceed more efficiently.

Other than deep learning-based software, scikit-image (van der Walt et al., 2014), ImageJ (Abràmoff et al., 2004), and PlantCV (Gehan et al., 2017) are also available for image processing and are broadly used in many applications (e.g., plant detection and leaf segmentation). Scikit-image and ImageJ require users to set thresholds manually for multiple color spaces to segment leaf areas. Therefore, if an image consists of various phenotypes of plants, for example, plants with green and red leaves due to some stress, simultaneously segmenting both plants may be challenging. PlantCV supports building task-specific machine learning models for instance segmentation. However, it does not support GUI and requires programming skills. Given these open-source packages, choosing the appropriate one for a task or a specific problem is often demanding. JustDeepIt is expected to address complicated issues and accelerate research on image analysis when used in combination with other software.

In plant science and agriculture, fruit detection and plant segmentation are two high-priority tasks (Arya et al., 2022; Zhou et al., 2022). This is because these tasks can estimate growth stages and yields of plants, including crops, and improve plant environmental robustness (e.g., disease resistance, fruit quality, and fruit yields) by collaborating with genomic technologies (e.g., genome-wide association study and expression quantitative trait locus analysis) (Varshney et al., 2021; Xiao et al., 2022). In this study, we represented the four case studies covering the two high-priority tasks with both GUI and CUI of JustDeepIt. The results support the robustness of JustDeepIt against critical issues in plant science. In addition, although JustDeepIt was intended for plant research, it may be applicable for image analysis in various disciplines beyond plant science. Furthermore, in a future version, we will continue to update the software in response to user demand and the technology flow.

## Data availability statement

The original contributions presented in the study are publicly available. This data can be found here: https://github.com/biunit/JustDeepIt.

## Author contributions

JS conceived the ideas and designed the software. JS and WC developed the software. JS, WC, and TY wrote the manuscript. All authors have read and approved the final manuscript.

## Funding

This study was supported by a research project of the Ministry of Agriculture, Forestry, and Fisheries and a Public/Private R&D Investment Strategic Expansion Program (PRISM) of the cabinet office of Japan, and JSPS KAKENHI Grant Number 22H05179.

## Acknowledgments

Computations were partially performed on the SHIHO supercomputer at the National Agriculture and Food Research Organization (NARO), Japan.

## Conflict of interest

The authors declare that the research was conducted in the absence of any commercial or financial relationships that could be construed as a potential conflict of interest.

## Publisher’s note

All claims expressed in this article are solely those of the authors and do not necessarily represent those of their affiliated organizations, or those of the publisher, the editors and the reviewers. Any product that may be evaluated in this article, or claim that may be made by its manufacturer, is not guaranteed or endorsed by the publisher.
